# Supplementary figures and images for: EHD2 overexpression promotes tumorigenesis and metastasis in triple-negative breast cancer by regulating store-operated calcium entry
Source: eLife. 2023 Jan 10;12:e81288. doi: 10.7554/eLife.81288 (PMC9988264; doi:10.7554/eLife.81288)

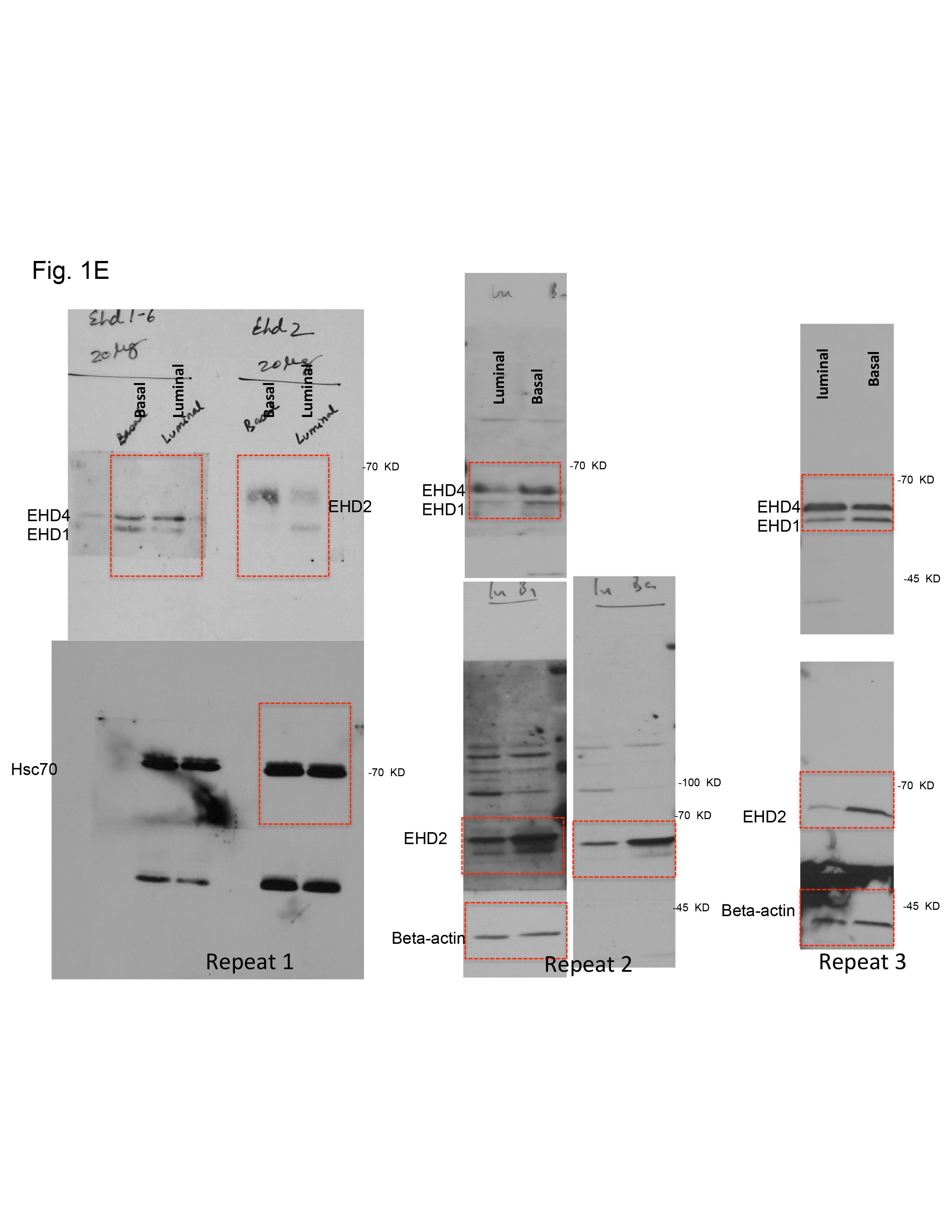

Supplement: Figure 1—source data 2. [file elife-81288-fig1-data2.tif]

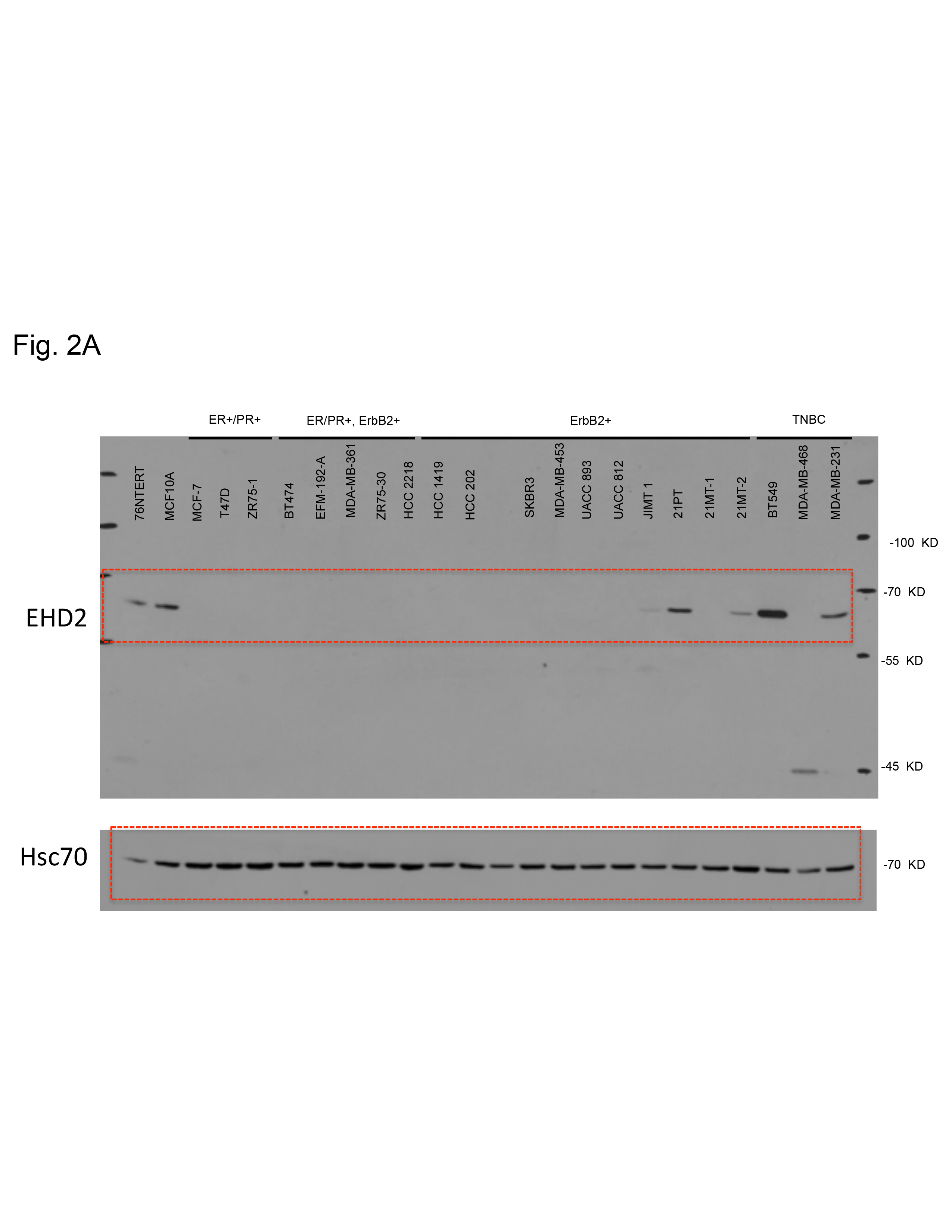

Supplement: Figure 2—source data 1. [file elife-81288-fig2-data1.tif]

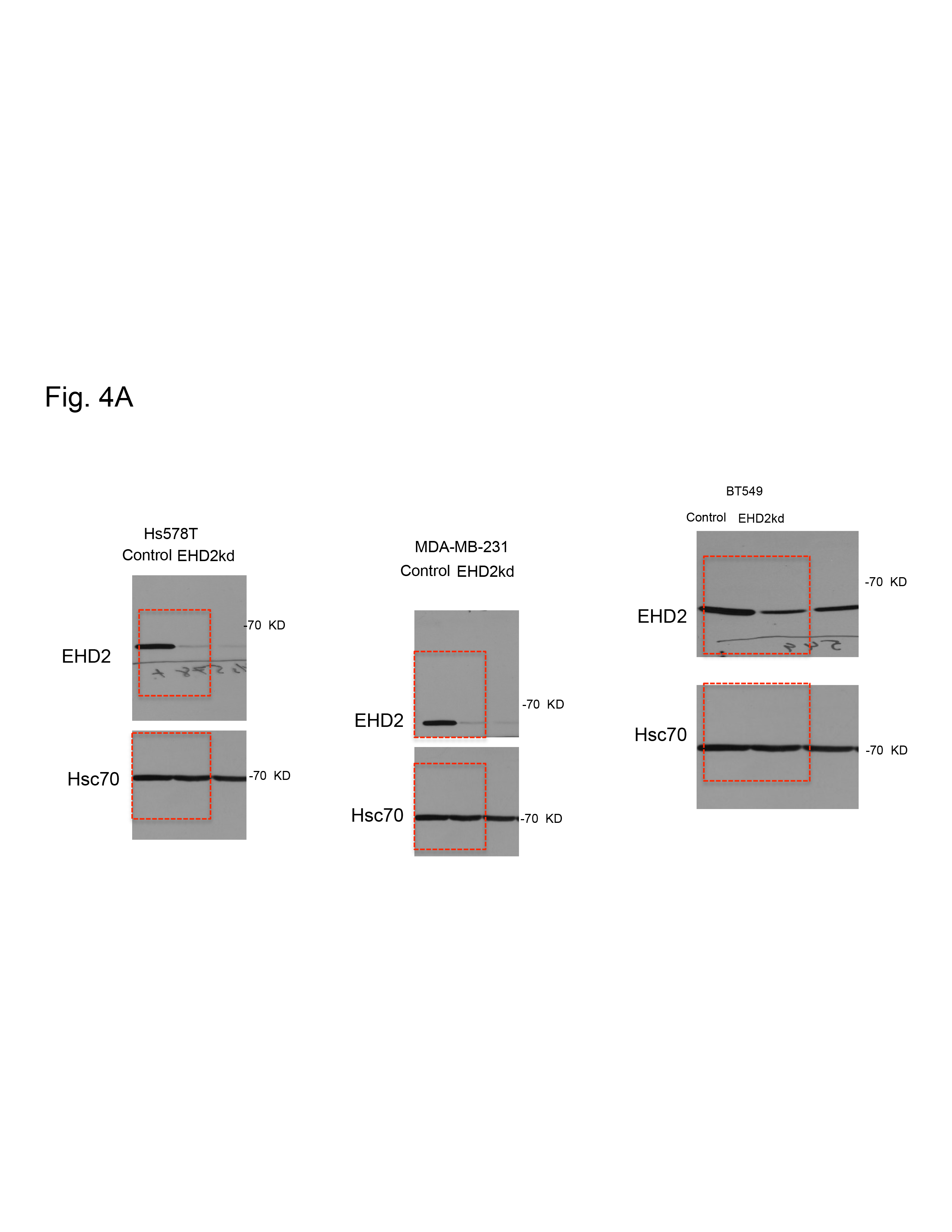

Supplement: Figure 4—source data 1. [file elife-81288-fig4-data1.tif]

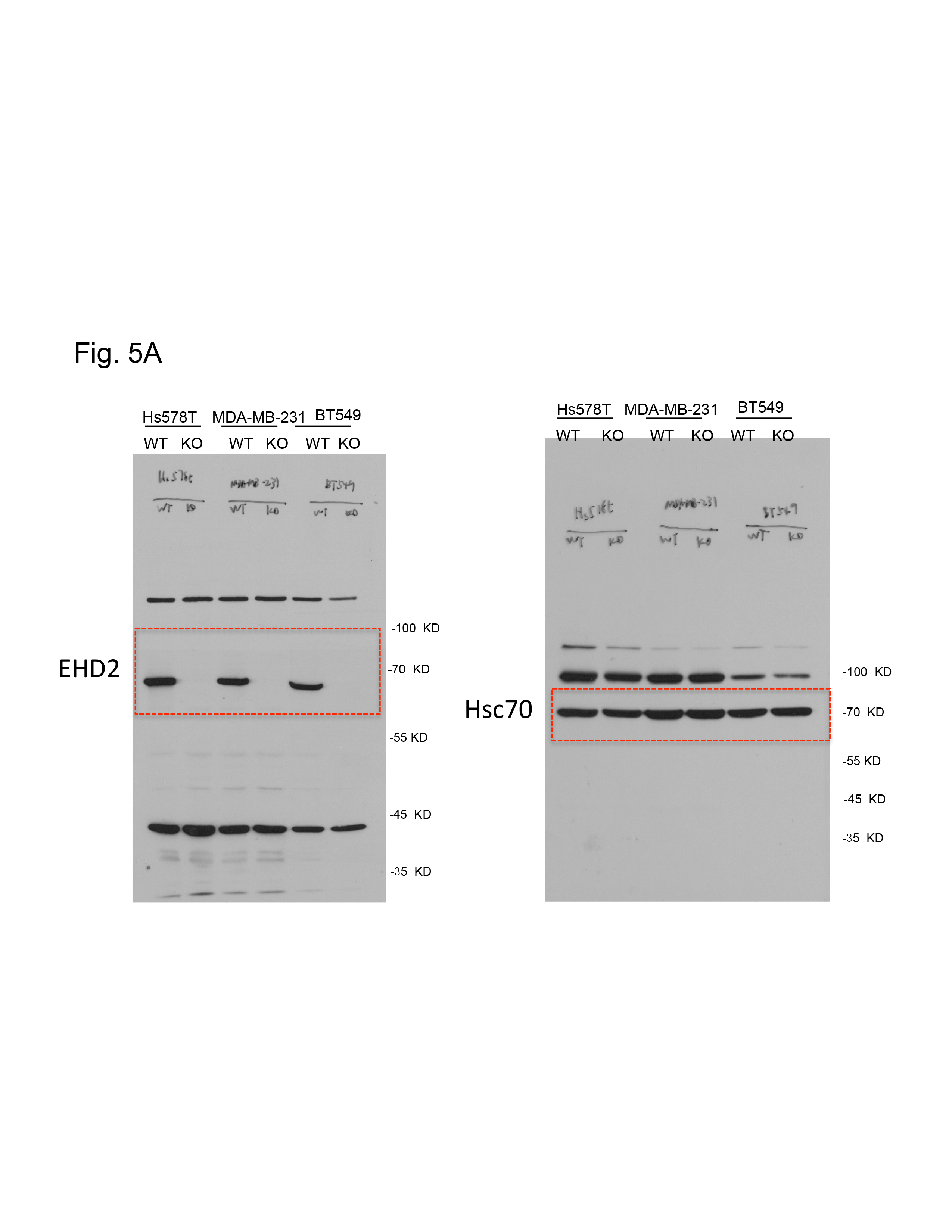

Supplement: Figure 5—source data 1. [file elife-81288-fig5-data1.tif]

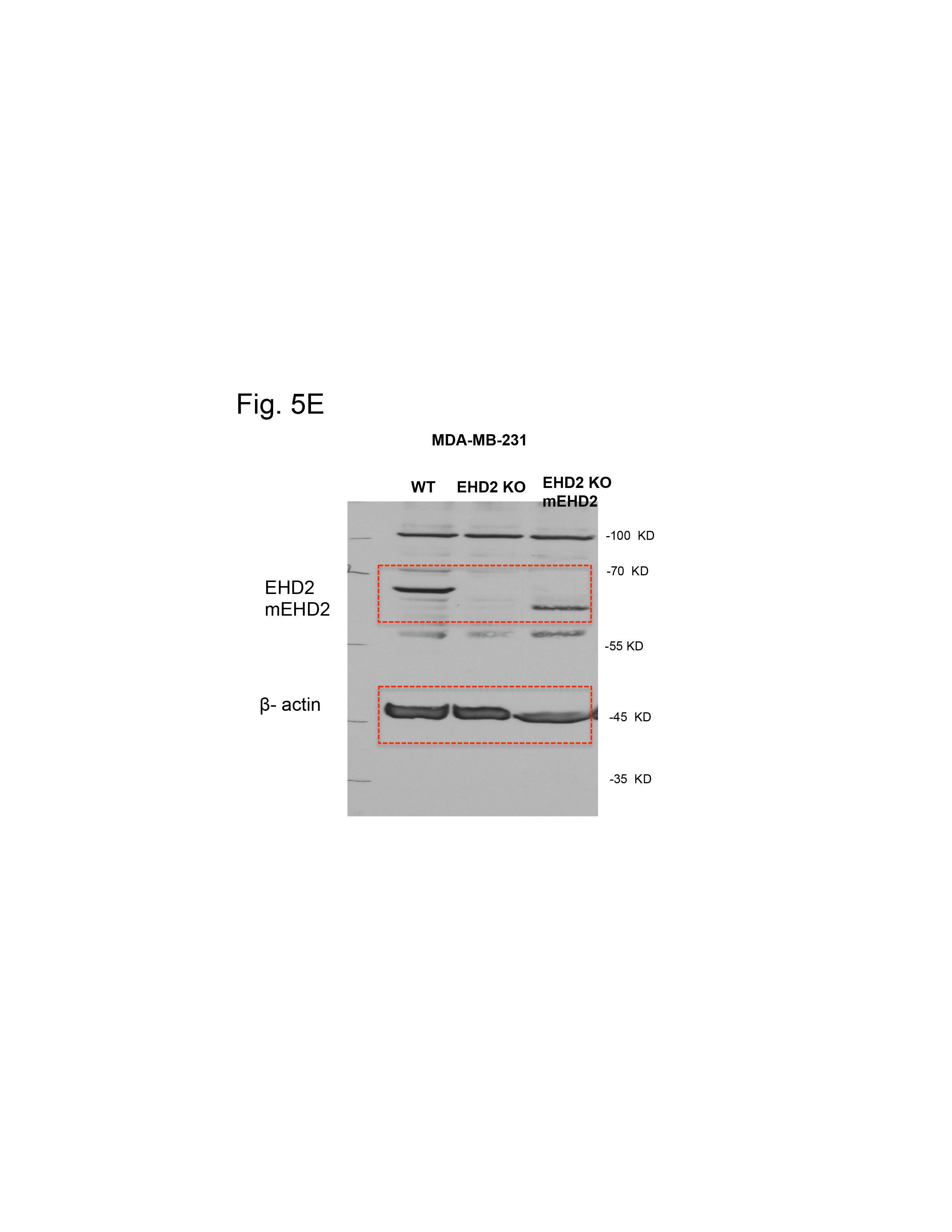

Supplement: Figure 5—source data 2. [file elife-81288-fig5-data2.tif]

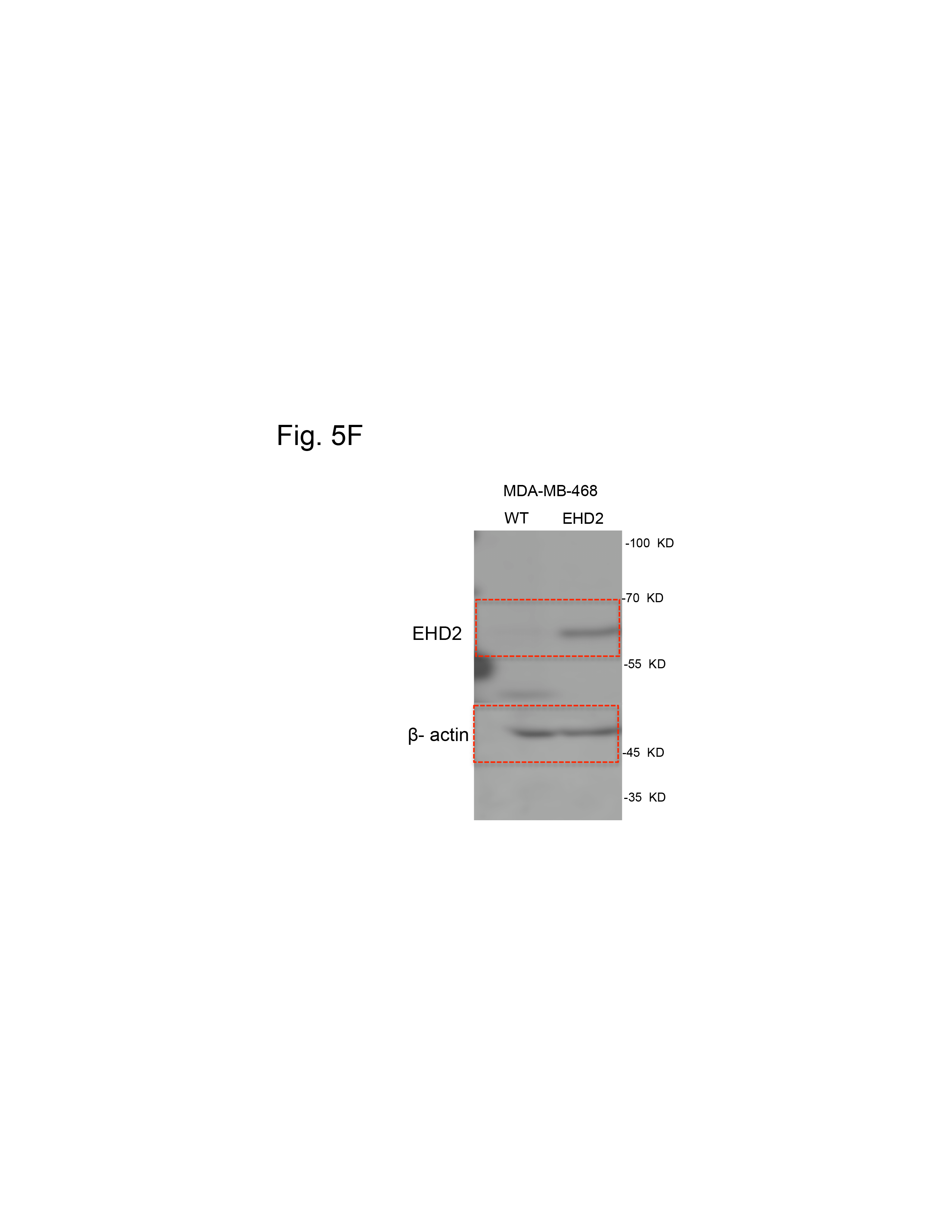

Supplement: Figure 5—source data 3. [file elife-81288-fig5-data3.tif]

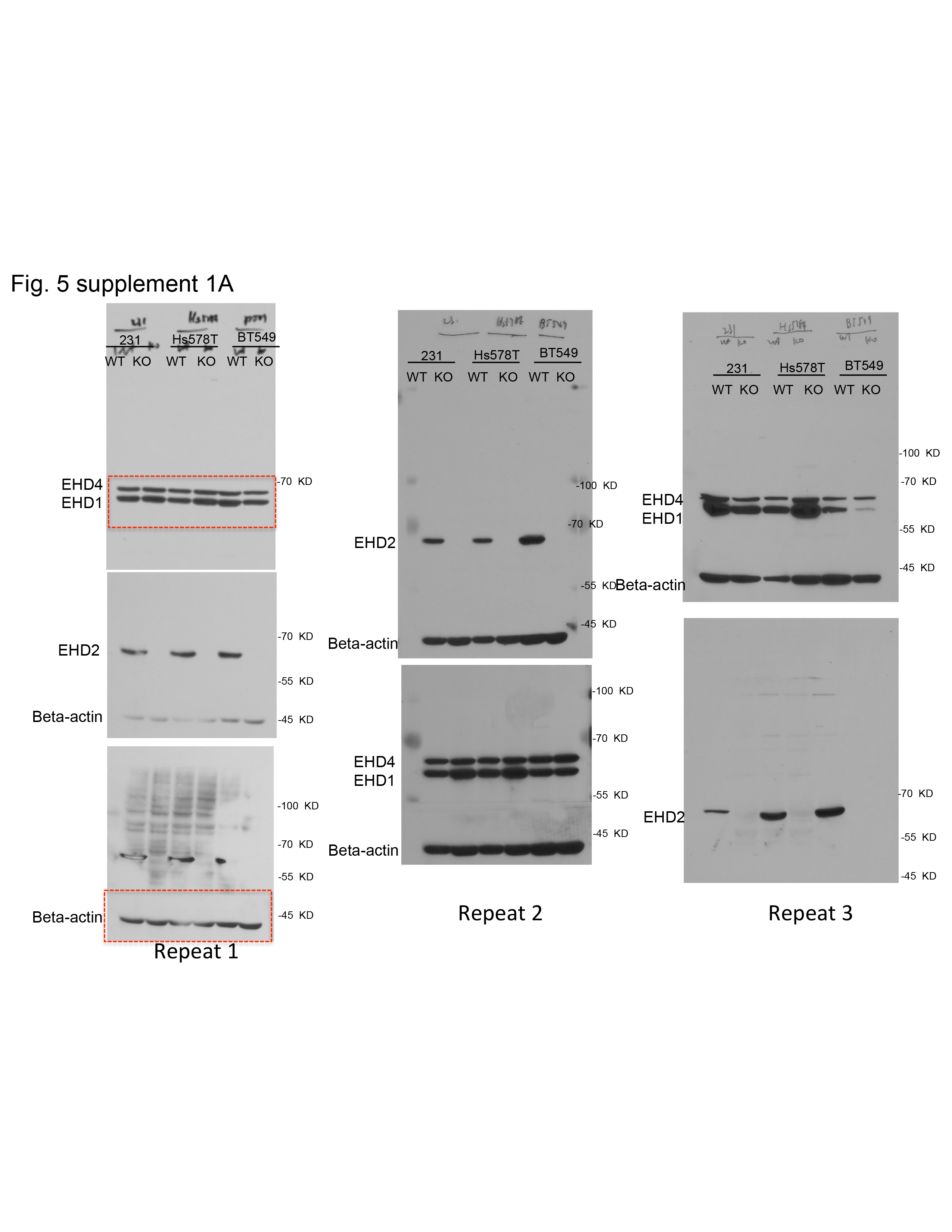

Supplement: Figure 5—figure supplement 1—source data 1. [file elife-81288-fig5-figsupp1-data1.tif]

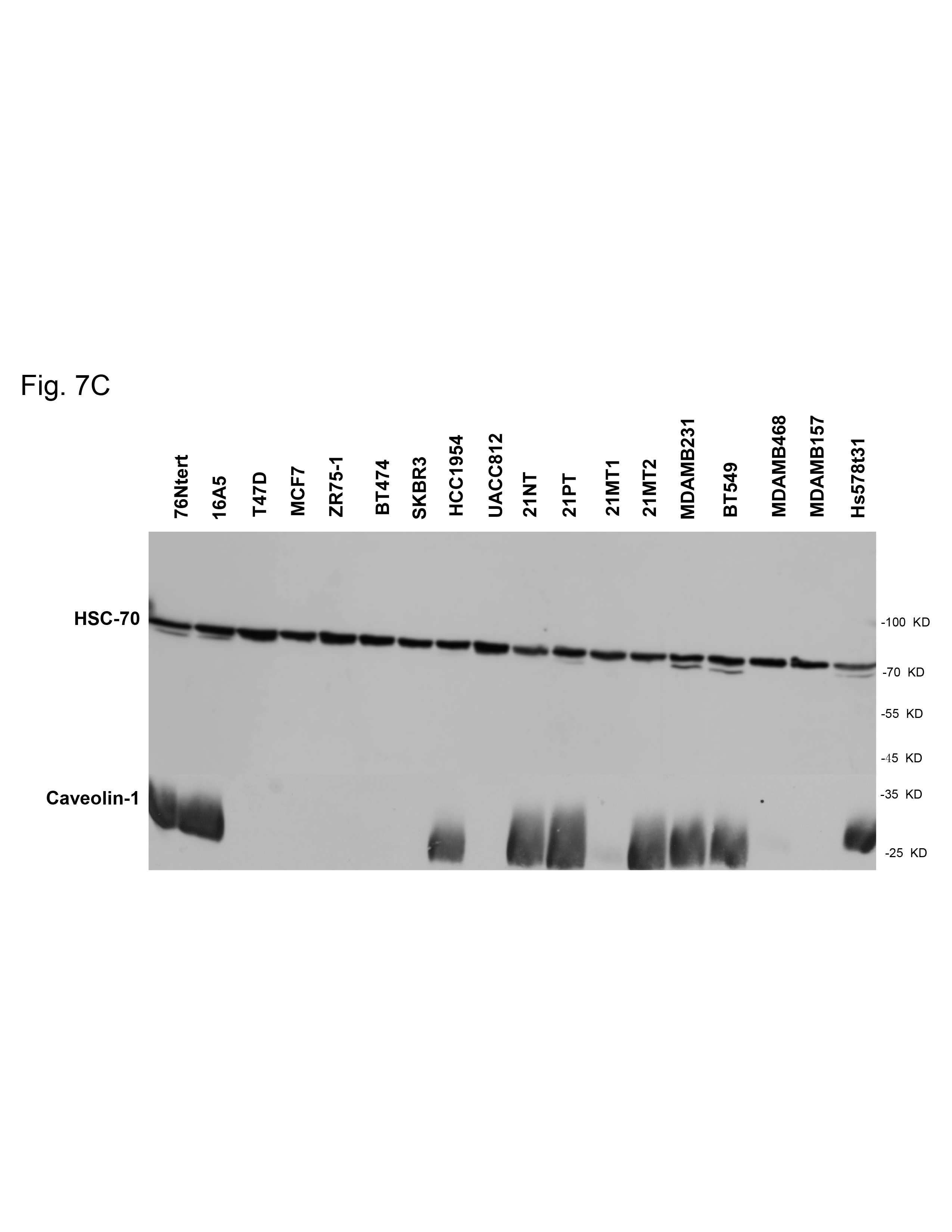

Supplement: Figure 7—source data 1. [file elife-81288-fig7-data1.tif]

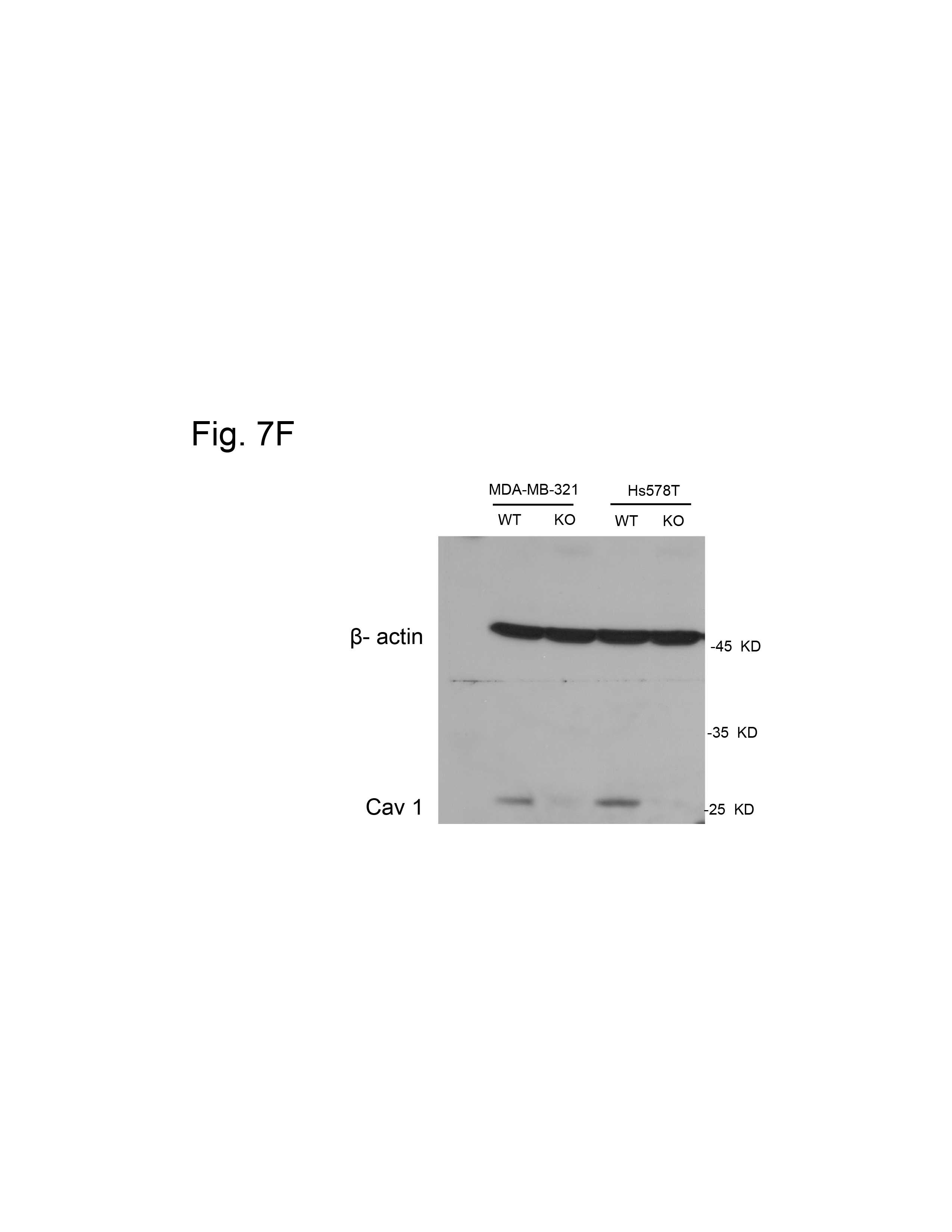

Supplement: Figure 7—source data 2. [file elife-81288-fig7-data2.tif]

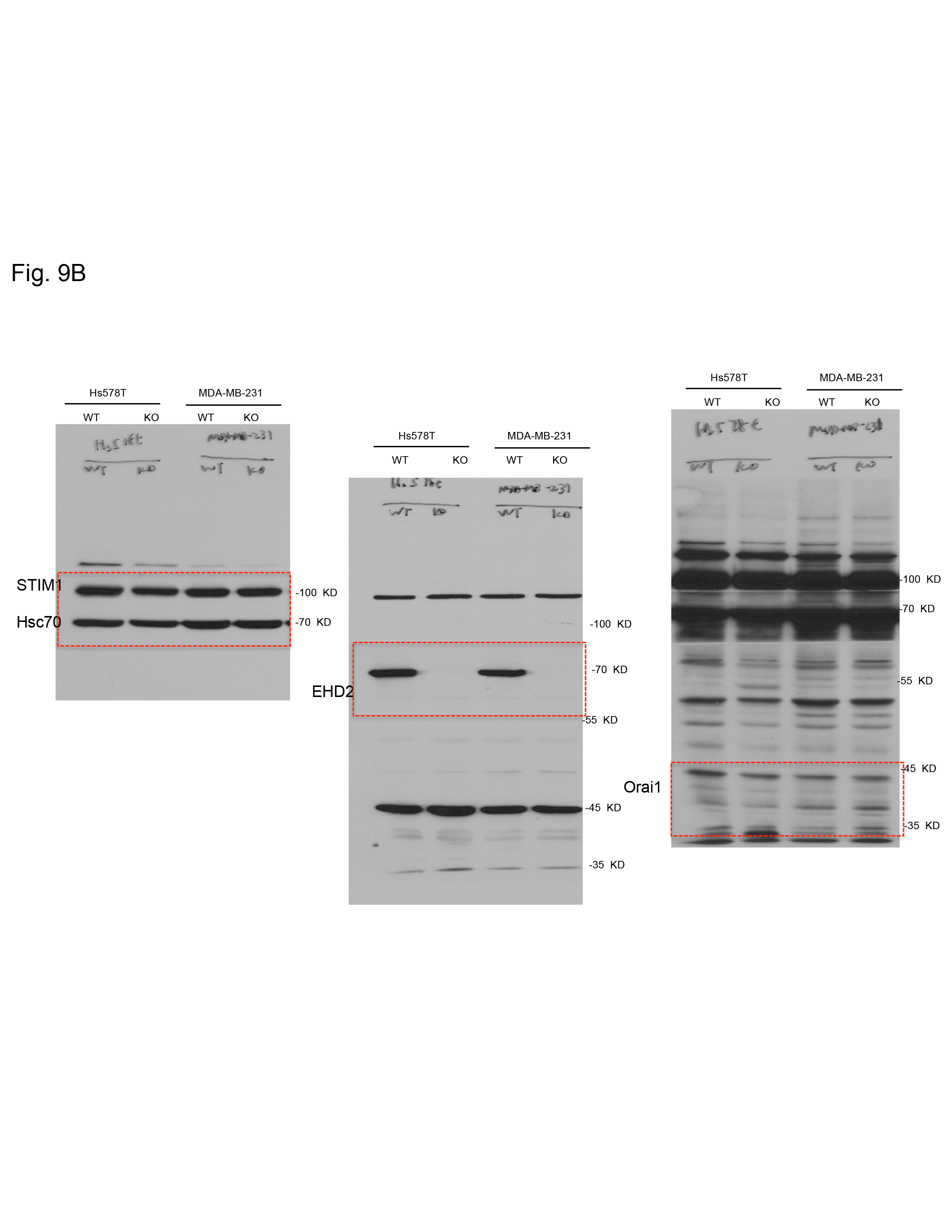

Supplement: Figure 9—source data 1. [file elife-81288-fig9-data1.tif]

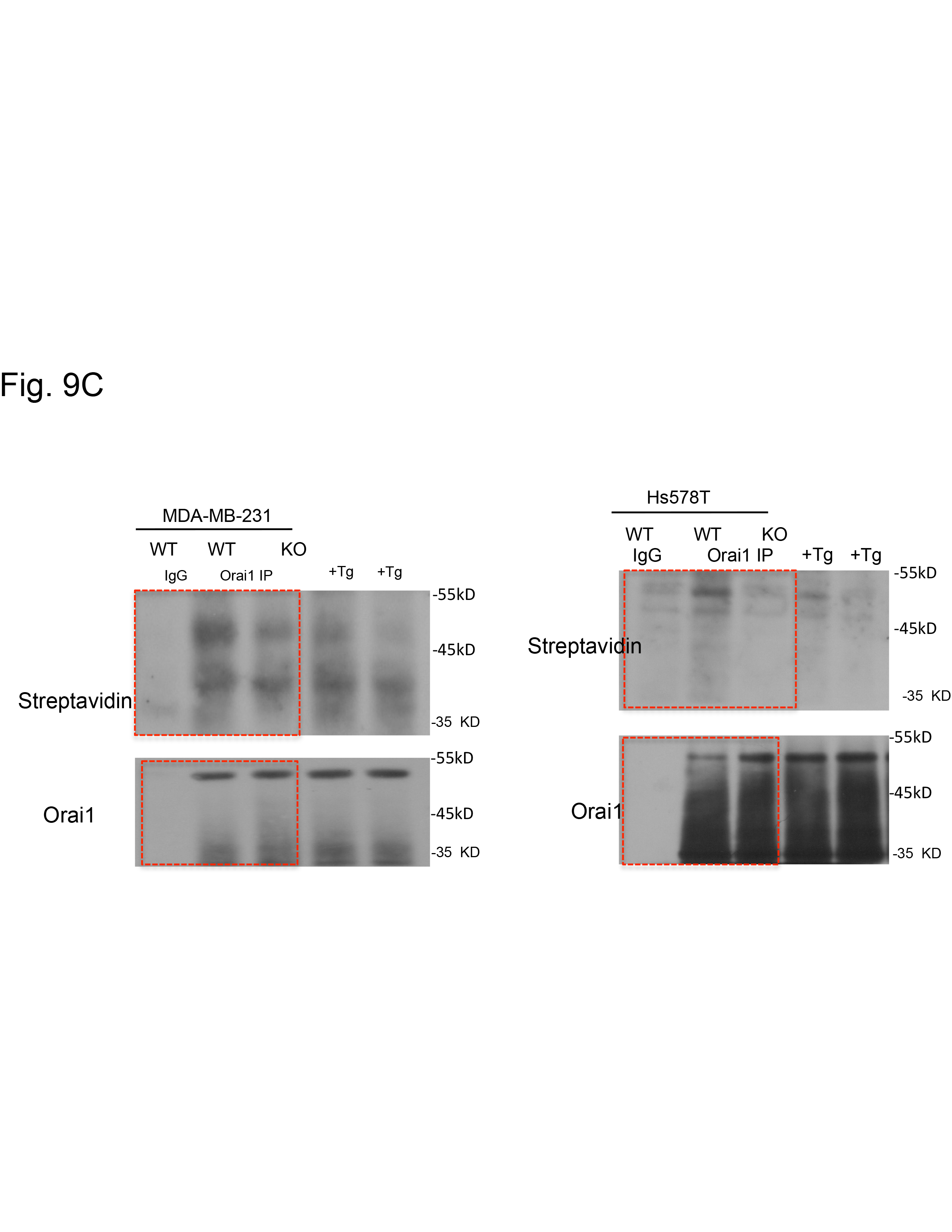

Supplement: Figure 9—source data 2. [file elife-81288-fig9-data2.tif]

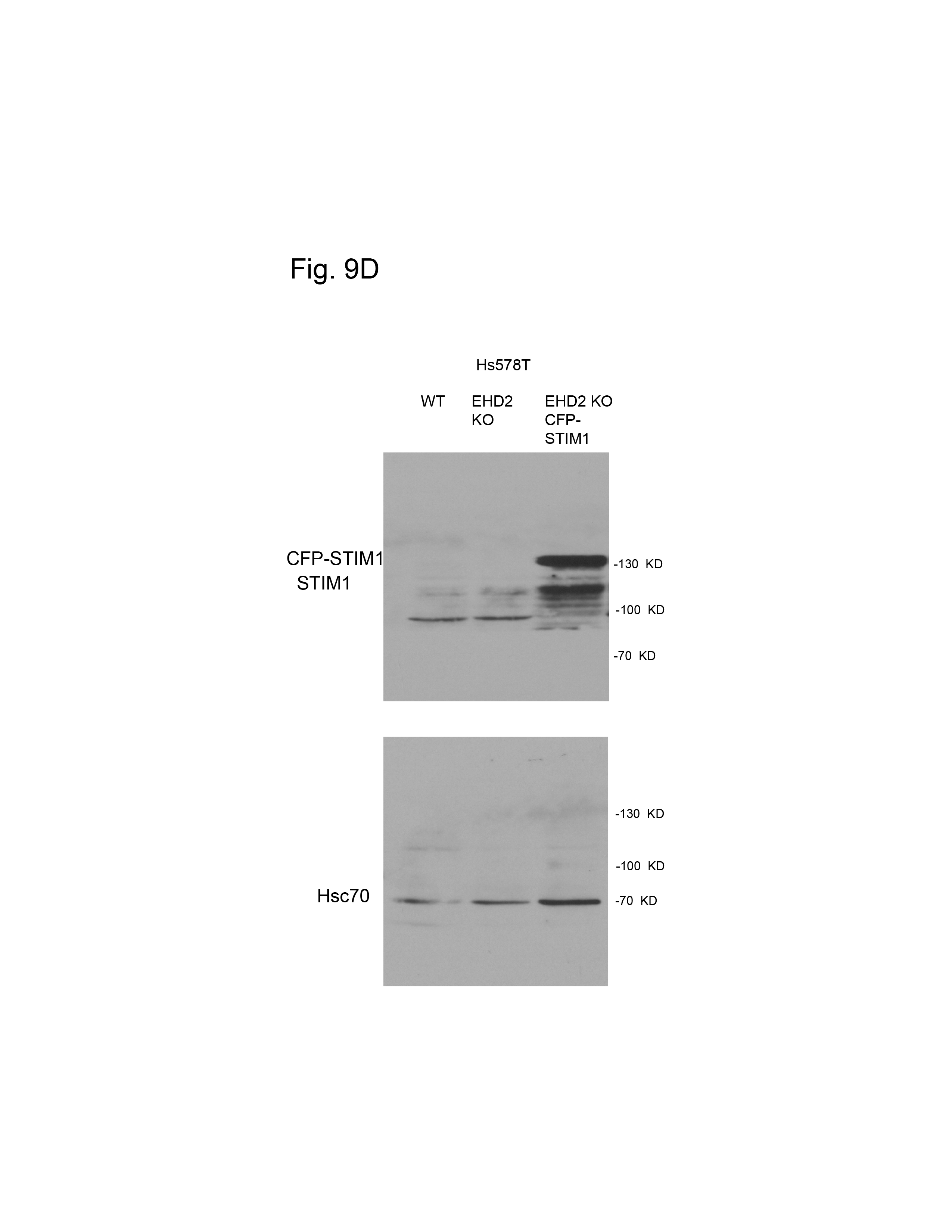

Supplement: Figure 9—source data 3. [file elife-81288-fig9-data3.tif]

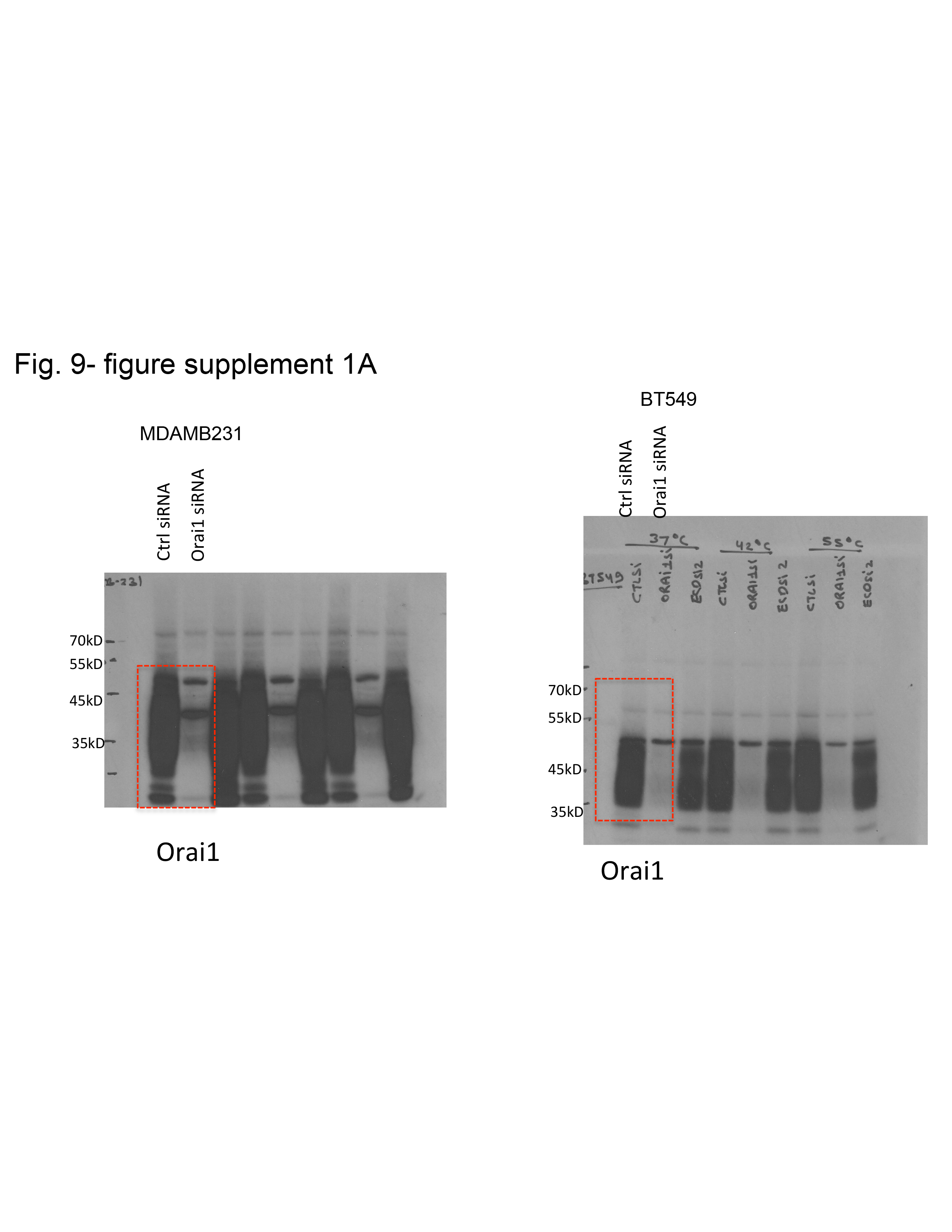

Supplement: Figure 9—figure supplement 1—source data 1. [file elife-81288-fig9-figsupp1-data1.tif]
